# Splenic injury following endoscopic drainage of a large pancreatic pseudocyst: a case report

**DOI:** 10.1186/s13256-021-03004-z

**Published:** 2021-08-15

**Authors:** Krittin J. Supapannachart, Christopher R. Funk, Lauren M. Gensler, Matthew P. Butters

**Affiliations:** 1 Emory University School of Medicine, 100 Woodruff Circle, 30322 Atlanta, Georgia; 2 Department of Medicine, Emory University School of Medicine, 100 Woodruff Circle, 30322 Atlanta, Georgia

**Keywords:** Pancreatic pseudocyst, Endoscopic drainage, Mass effect, Splenic injury, Case report

## Abstract

**Background:**

Many pancreatic pseudocysts spontaneously resolve, but larger or symptomatic pseudocysts may require procedural management. Though endoscopic ultrasound guided approaches are standard of care and have high success rates, complications can include bleeding, infection, and splenic perforation. This patient case report details an unusual series of complications of endoscopic cystogastrostomy that should encourage clinicians to evaluate for anatomic disruptions caused by mass effects of pancreatic pseudocysts prior to endoscopic pseudocyst drainage.

**Case presentation:**

A 53-year-old African American male with a past medical history notable for alcohol use disorder, chronic pancreatitis, and insulin dependent diabetes presented with a 4-day history of left upper quadrant abdominal pain. Computed tomography imaging with contrast revealed enlargement of a known pancreatic pseudocyst to 15.9 × 10.4 cm. Due to pseudocyst size and the patient’s symptoms, endoscopic cystogastrostomy stent placement was performed. However, postprocedurally, he developed leukocytosis to 19,800 cells/m^3^ (from 14,100 cells/m^3^ preoperatively) as well as acute hypoxemic respiratory failure with a large left pleural effusion. Postprocedural computed tomography with contrast demonstrated a new large subcapsular splenic hematoma in communication with a new subdiaphragmatic fluid collection. Due to suspicion of endoscopic procedural complication, he underwent open laparotomy which revealed grade 4 splenic laceration, septic splenic hematoma, and a subdiaphragmatic abscess.

**Conclusions:**

While endoscopic drainage of pancreatic pseudocyst was technically successful, this case demonstrates complications from mass effect of a large pancreatic pseudocyst which putatively tore the splenorenal ligament, leading to excessive separation of the left kidney and spleen. If anatomic disruptions caused by mass effect from a pancreatic pseudocyst are recognized through preprocedural abdominal imaging, such cases may be considered for early open repair versus cystogastrostomy.

## Background

Pancreatic pseudocysts are a well-established complication of acute and chronic pancreatitis [1]. While up to 50% of pseudocysts spontaneously resolve, those that remain persistent can cause significant symptoms necessitating procedural intervention [2]. The current standard of care for pancreatic pseudocysts is through endoscopic and ultrasound (EUS)-guided transmural or transpapillary approach, which allows for insertion of a stent to drain cyst contents through the gastrointestinal tract [3, 4]. Though this approach has been documented to have success rates of about 90%, the procedure is also associated with substantial complications such as bleeding, infection, and perforation in up to 37% of patients [5–7]. Some complications, such as pseudoaneurysm rupture, can be avoided through appropriate preoperative imaging [8]. Here, we present the case of a large pancreatic pseudocyst that caused upward displacement of the spleen from the left kidney and upon pseudocyst drainage led to development of a large subcapsular hematoma and splenic abscess, necessitating open repair.

## Case presentation

**Fig. 1:**
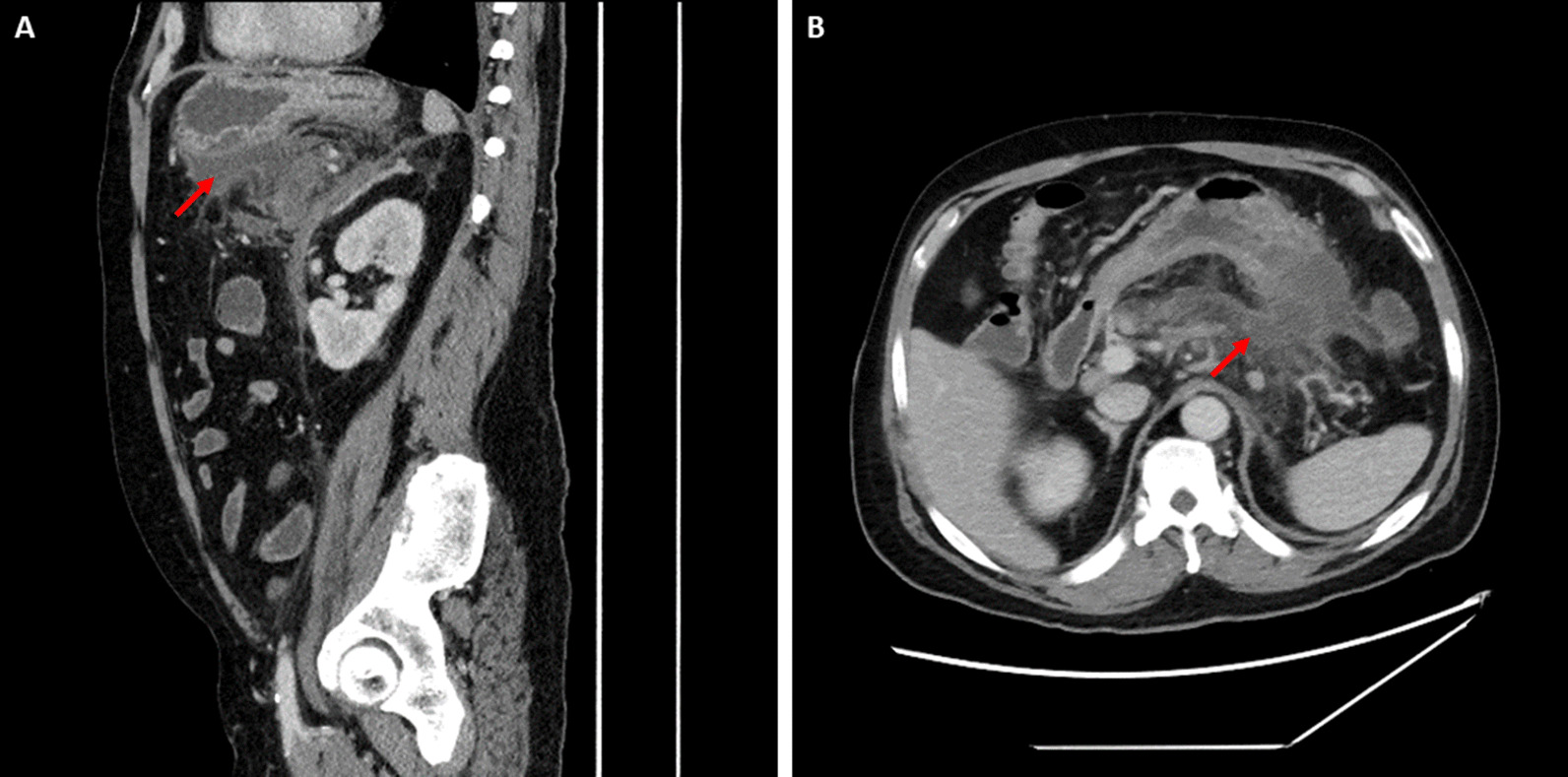
Computed tomography imaging of the abdomen and pelvis with intravenous contrast of a pancreatic pseudocyst on initial discovery (7.1 × 2.8 × 8.8 cm). **A** Sagittal view, **B** axial view. Red arrows denote pseudocyst

A 53-year-old African American male with a past medical history notable for chronic pancreatitis, insulin-dependent diabetes, hypertension, peripheral arterial disease, and gout presented to the emergency room complaining of acute left upper quadrant abdominal pain. At baseline, he was fully functional, has held office jobs, smoked a few cigarettes per day over the course of a few years but quit over 5 years prior to initial presentation, and reported drinking around four alcoholic beverages daily. Home medications include insulin, metformin 500 mg daily, lisinopril 40 mg daily, amlodipine 10 mg daily, metoprolol 25 mg twice per day, rosuvastatin 40 mg per day, aspirin 81 mg, clopidogrel 75 mg per day, and allopurinol 100 mg. Family history was notable for insulin-dependent diabetes and hypertension in his mother and hypertension in his father. Eighteen months prior to the current presentation, he presented to the emergency room with postprandial abdominal pain. Physical examination revealed an uncomfortable but alert patient who was tachycardic to 110 beats per minute and had decreased breath sounds at the bilateral lung bases. Abdomen was nontender but mildly distended. Neurologic examination was unremarkable. Computed tomography (CT) imaging of the abdomen was notable for necrotizing acute pancreatitis and a fluid collection between the pancreas and stomach measuring 7.1 × 2.8 × 8.8 cm (Fig. 1). He was managed supportively and discharged. A few months later, he was admitted for diabetic ketoacidosis secondary to pancreatic necrosis; no abdominal imaging was obtained at that visit. Repeat CT abdomen 5 months after the initial episode, or 2 months after his diabetic ketoacidosis admission, revealed no change in size of the pancreatic pseudocyst (not shown).

**Fig. 2:**
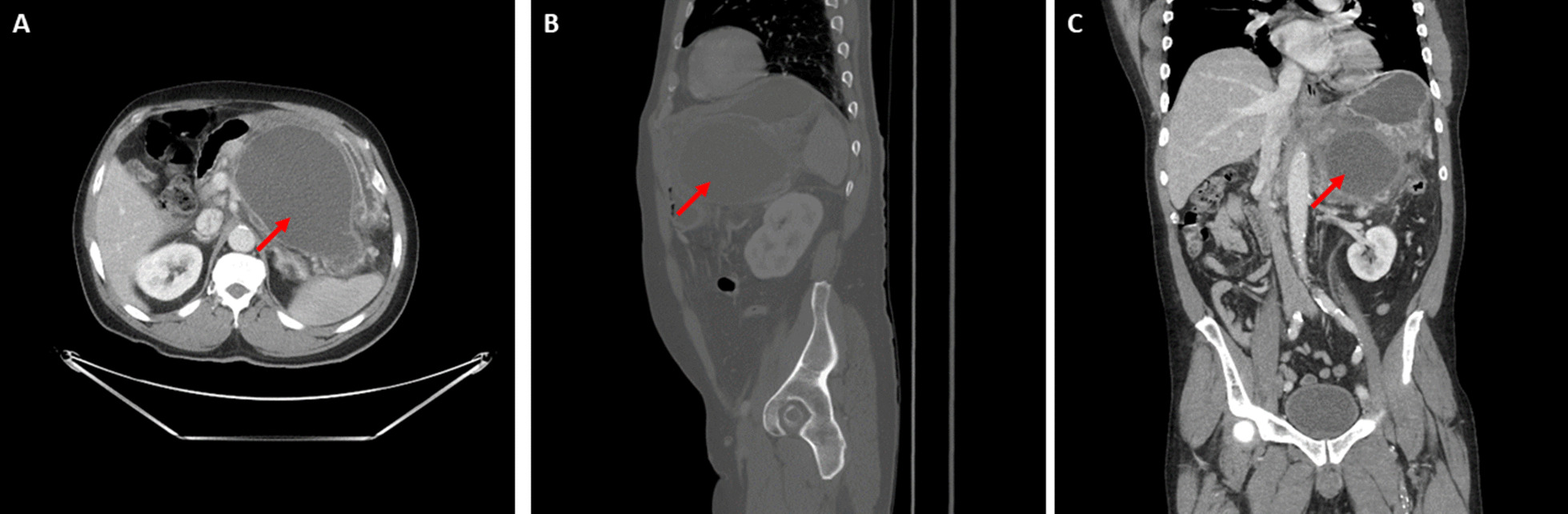
Computed tomography imaging of the abdomen and pelvis with intravenous contrast of a pancreatic pseudocyst on admission (15.9 × 10.4 cm). **A** Axial view, **B** sagittal view. **C** Coronal view. Red arrows denote the pseudocyst

On the current presentation, the patient presented with 4 days of postprandial, sharp abdominal pain with radiation to his back. The patient stated the pain was of different character than the original episode of acute pancreatitis 18 months prior but was unable to specifically describe how. Vital signs were temperature of 37.4 °C, blood pressure of 161/88 mmHg, heart rate of 90 beats per minute, and O_2_ saturation 100% on room air. White blood cell count was 14.7 × 10^3^ cells/m^3^, hemoglobin 16.7 g/dl, glucose of 221 mg/dl, and HgbA1C of 11.3%. His serum bicarbonate was 23 mEq/l, and he did not have diabetic ketoacidosis. Serum lipase was 145 U/L. Other laboratory values, including electrolytes, renal function, and liver function tests, were unremarkable. CT abdomen revealed enlargement of the pseudocyst to 15.9 × 10.4 cm (Fig. 2), which displaced the stomach and compressed the splenic vasculature. Home medications except clopidogrel were continued during admission. The patient also received intravenous 0.9% normal saline maintenance fluid at 100 mL per hour infusion rate, oral acetaminophen 650 mg every 6 hours for pain, oral Roxicodone 5 mg every 4 hours as needed for severe pain, and intravenous morphine sulfate 4 mg every 4 hours as needed for breakthrough pain. After conferring with gastroenterology, endoscopic cystogastrostomy was performed with 1.1 L of fluid drained from the cyst during the procedure, and a cystogastrostomy stent was placed to drain the remaining fluid.

Immediately following extubation from the endoscopic cystogastrostomy, he developed respiratory distress: O_2_ saturation was 93% on 15 L non-rebreather. He developed new left-sided flank pain that was exquisitely tender to palpation. Leukocytosis worsened to 19.8 × 10^3^ cells/m^3^. Chest radiograph demonstrated a new moderately sized left-sided pleural effusion without consolidation, and additional CT chest revealed new left-flank fat stranding. Prophylactic antibiotics, oral levofloxacin 750 mg daily and intravenous metronidazole 500 mg every 8 hours, were given. Thoracentesis was performed, which drained 700 mL of fluid, but the pleural effusion size remained unchanged on serial imaging, suggesting reaccumulation. Pleural fluid analytes were lactate dehydrogenase 591 U/L, amylase 115 U/L, glucose 161 mg/dL, pH 7.58, 10,386 red blood cells/μL, and 3088 white blood cells/μL. When compared with serum chemistry, the effusion was exudative and the etiology was thought to be sympathetic pleural effusion secondary to his pancreatitis. Hemothorax secondary to splenic hematoma formation was thought to be less likely given the serosanguinous appearance of drainage and the relatively low red blood cell count. However, a hematocrit value was not obtained to definitively rule this out.

**Fig. 3:**
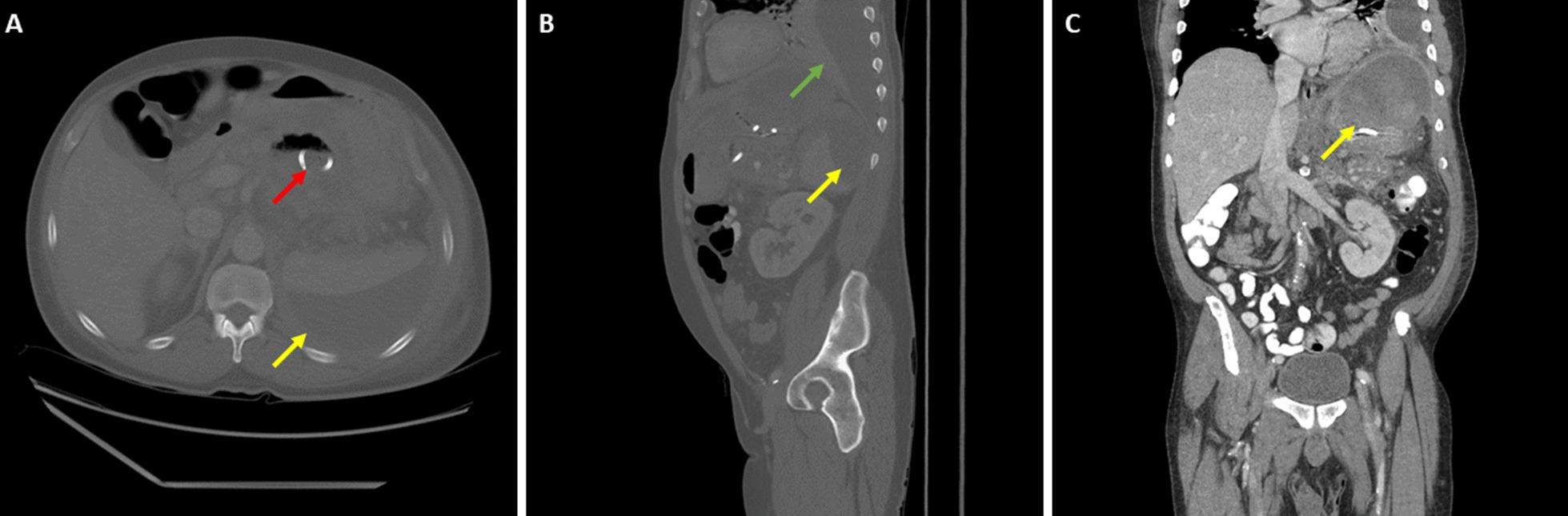
Computed tomography imaging of the abdomen and pelvis with intravenous contrast of pancreatic pseudocyst and new subcapsular collection after cystogastrostomy placement. **A** Axial view, **B** sagittal view, **C** coronal view. Red arrow denotes cystogastrostomy tube, yellow arrows denote subcapsular collection, and green arrow denotes new left-sided pleural effusion

Due to concern for a pancreaticopleural fistula, further CT chest and abdomen were obtained. CT abdomen demonstrated decreased size of the original pseudocyst and appropriate placement of the cystogastrostomy stent but also noted a new large left subdiaphragmatic fluid collection communicating with a large subcapsular splenic hematoma (Fig. 3). The hematoma was absent from prior imaging. A second CT chest revealed no sign of communication between the pleural effusion and subdiaphragmatic collections.

He was taken for exploratory laparotomy and splenectomy as there was suspicion of septic splenic hematoma due to fever and leukocytosis. Intraoperatively, significant inflammation leading to obliteration of the lesser omental sac and malrotation of the spleen were noted. Gross inspection of the spleen revealed a 300 mL splenic abscess, grade IV splenic laceration that led to formation of a large subcapsular splenic hematoma. Pathology further noted two disruptive areas adjacent to the splenic hilum measuring 6.0 × 1.0 cm and 5.0 × 1.0 cm. The pleural effusion was subsequently drained via tube thoracostomy and resolved 2 weeks prior to discharge. The patient was discharged after protracted postoperative course. He was followed as an outpatient after discharge with no surgical complications at 7 months or changes to his medication regimen.

## Discussions and conclusions

Subcapsular splenic hematomas are a known but rare complication of pancreatic pseudocyst treatment. This case report illustrates an atypical mechanism for their formation and advocates for careful preoperative review of anatomical structures around large pancreatic pseudocysts prior to drainage [9].

Splenic hematomas in the setting of pancreatitis typically form from pancreatic enzyme leakage eroding the spleen itself or disruption of the splenic vessels [10]. Another case report demonstrates that splenic rupture can also occur through pancreatic enzyme leak when pancreatic pseudocysts come into contact with the splenic parenchyma; it does not have to be during an episode of acute pancreatitis [11]. Additionally, splenic vein thromboses have been demonstrated as a contributory factor in splenic rupture or hematoma formation among patients with pancreatic pseudocysts; irregularities in the splenic vein were also noted on CT imaging of this patient but could not be further characterized [12]. Based on discussions between the medicine team, radiology, general surgery, and gastroenterology, the presumptive mechanism of hematoma formation in the current case was pseudocyst expansion leading to tearing of the splenorenal ligament and excessive separation of the left kidney and spleen. While this was not an issue prior to drainage as the pseudocyst was acting as structural support for the spleen, drainage of the pseudocyst caused the spleen to float freely in the abdomen and become prone to vascular compromise secondary to torsion. It is still possible, however, that another mechanism better explains the observed splenic injuries. Even though anatomical separation of the left kidney and spleen is observed on CT imaging, splenorenal ligament tearing was not confirmed intraoperatively.

As for the pleural effusion, pulmonology thought the etiology was sympathetic effusion caused by increased vascular permeability rather than a diaphragmatic defect or esophageal rupture given the pleural fluid laboratory findings and lack of observable diaphragmatic defects on repeat CT imaging. Prior case reports have described patients with pancreatic pseudocysts presenting with pleural effusions due to pancreaticopleural fistula formation; however, to our knowledge, there are no reports detailing new-onset pleural effusions immediately following endoscopic drainage [13]. The timing of the pleural effusion in this case may imply a procedural complication occurred, such as formation of a small diaphragmatic defect or a hemothorax.

In conclusion, it may be beneficial to evaluate patients with large and symptomatic pancreatic pseudocysts for splenic complications, even when they do not initially present with signs of splenic involvement such as left-sided flank pain or definitive imaging revealing splenic injury or splenic vascular compression. Careful consideration of the anatomical disruptions caused by enlargement of the pseudocyst may prevent severe complications of pseudocyst drainage by altering management decisions. In this case, knowledge of potential tearing of the splenorenal ligament would have prompted early surgical consideration for management rather than endoscopic cystogastrostomy.

## Data Availability

Not applicable.
